# Supplementary material for: Metabolomics and Transcriptomics Analyses Uncover the Potential of Flavonoids in Response to Saline–Alkali Stress in Codonopsis pilosula
Source: Biology (Basel). 2025 Dec 9;14(12):1759. doi: 10.3390/biology14121759 (PMC12730329; doi:10.3390/biology14121759)
Supplement: Supplementary file 1 [file biology-14-01759-s001.zip › Figure S1.pdf]

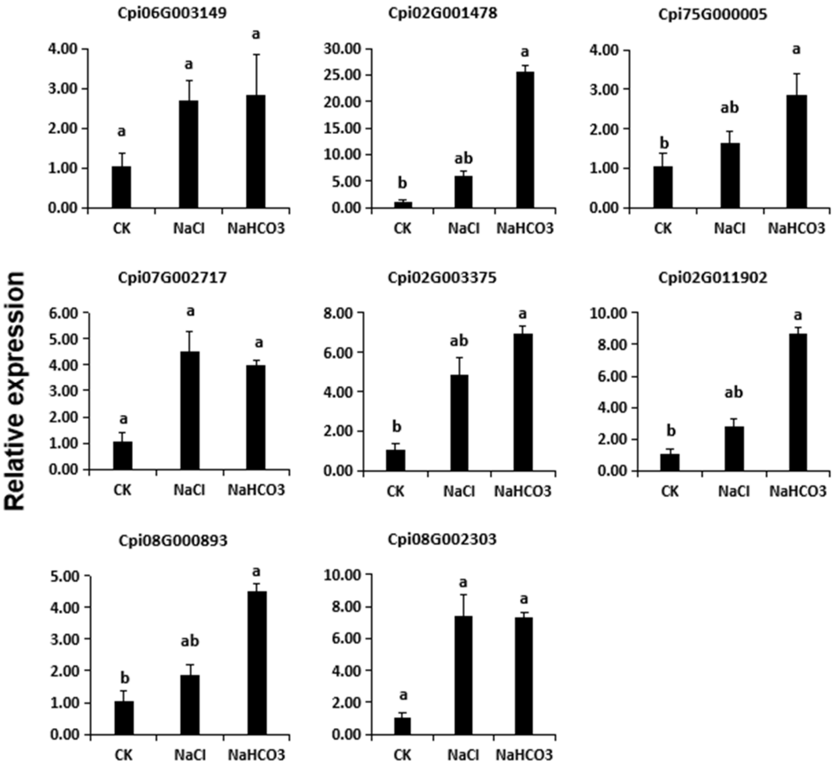

Figure S1. RT-qPCR detection of DEGs of Cp. Columns marked by the same lowercase letters are not significantly different according to Duncan's multiple range test (P< 0.05).
